# Collectanea Medica: Consisting of Anecdotes, Facts, Extracts, Illustrations, &c.

**Published:** 1820-10

**Authors:** 


### COLLECTANEA MEDICA:

CONSISTING OF

### ANECDOTES, FACTS, EXTRACTS, ILLUSTRATIONS, &c.

*Relating to the History or the Art of Medicine, and the Auxiliary Sciences.*

Floriferis ut apes in saltibus omnia libant,

Omnia nos itidem depascimur aurea dicta.

### *Extracts of the Report from the Select Committee of the House of Commons on the Doctrine of the Contagion of the Plague, in* 1819.†

*Veneris,* 19° *die Martij,* 1819.

SIR JOHN JACKSON, Baronet, in the Chair.

SIR JAMES M'GREGOR, called in; and Examined.

YOU have been long in the medical profession?—I have been on the medical staff of the army twenty-six years.

Are you acquainted with the plague?—I have seen the plague in Egypt, and am (as director-general of the army medical department) in possession of the details of the plague in Malta and the Ionian Isles.

† We select only the accounts of the examinations of those physicians who have had opportunities for personal observation of the Plague; the whole of which we shall adduce, and in detail, excepting that of Dr. CHARLES MACLEAN, which we omit solely because this physician's opinions were previously well known to the profession in general, by the medium of his writings on this subject.—EDIT.

In what year was you in Egypt?—I went there in 1801.

Were there a great many cases of plague that came under your own inspection?—Few under my own inspection. I was at the head of the medical department of the army which came from India to Egypt; it was my duty to apportion the attendance, and frame general arrangements.

Had you many cases of plague that came under your inspection?—Under my own immediate inspection: they were the first cases that appeared in the Indian army; there were 165 in that army.

Did the whole 165 come under your own personal inspection?—They did not.

But several of the cases came under your personal inspection: state to the Committee the circumstances attending them: the first case that came under your observation, was it attended with buboes?—With buboes.

With fever?—Fever and buboes.

You then considered it to be true plague?—Most undoubtedly. The two first cases that appeared were hospital servants.

Did the cases prove fatal?—These two, and four other men that slept in the room, all proved fatal.

How do you suppose the first patient caught it?—It never could be traced; but we discovered that the plague existed then in Rosetta, where hospitals were situate.

Do you suppose the others who were afterwards infected, caught it of them?—It was distinctly traced to those men. Perhaps I could make this matter clearer by referring to a statement which I published at the time.

Was the plague in the country at the time the two Indian servants caught it?—Yes: the hospital in which the first cases of plague appeared, viz. that of the 88th regiment, was situate in Rosetta, and the plague was there at the time.

Give your own account?—These cases and the subsequent ones had fever, followed by buboes; but, towards the end of the season, in April, May, and June, bubo was seen as the first, and in some cases as the only, symptom: the seasons from September till June; the first case appeared on 14th September, and the last in June.

Do you believe the plague to be contagious?—Certainly, by contact. I should have some doubts of a very close atmosphere, but I have no direct evidence of that; I could often trace it to contact.

Can it be taken by any other means or cause?—I should think most cases are by actual contact.

Not infection?—No, contact.

Do you mean actual contact with the body, or clothes infected with miasmata?—Both.

Do you think that the air has no operation in the production of disease?—There is a variety of opinion as to that point: a very general opinion is, that a particular constitution of the air is favourable to its spreading.

Then you consider the air as assisting?—Perhaps it may; but I am in possession of no facts to enable me to give a decided opinion on this part of the subject.

What is your reason for considering it contagious?—From having traced it clearly from one subject to another, on the first appearance of the disease in Egypt in 1801. In an hospital where there were 165 men, the disease appeared first in two native Indian servants; they were separated on the following day; but other four servants who slept in the same room, and who, I believe, slept in the same blankets, caught the disease, and the whole six died.

What reason have you to suppose the two first took it by contagion? —I could not immediately trace its origin in the two first cases; but subsequently it was traced from the two servants to all who were afterwards attacked.

Might it not have been produced by the original agency of the air? —There was nothing particular in the air of the hospital where the plague appeared, nor any thing that could be discovered different from all the other hospitals, or from the hospital to which the whole 150 men were removed afterwards. Perhaps it might be satisfactory to know that, in the regimental hospital in which it first appeared, it was got rid of by separation.

Separation from what?—After these six men were removed, others with symptoms of fever were immediately separated, and placed in distinct rooms under observation. As I have stated, there were about 165 men in that hospital: immediately after the discovery of the plague, all the cases of it were sent to a pest-house; the suspicious cases, those with symptoms of fever, were placed in observation-rooms; and then the whole of the remainder of the 165 men were, without loss of time, removed to another hospital prepared for them. Before any man was removed to the new hospital, the strictest precautions were used: his hair being cut off, he was put into a bath, and the whole of his clothing left outside the hospittal, and destroyed; each man was then provided with new and fresh clothing. The consequence of these precautions having been rigidly enforced was, that, after entering the new hospital, no new case of the disease appeared.

Were they infected with the plague before they went into the hospital?—No.

Do you mean, that all those who went into the hospital were persons not infected?—Not infected: care was taken to prevent any person with the slightest appearance of the disease from going there.

Do you think the plague can be produced from goods and things, as well as persons?—I should think from goods; I can speak with certainty of the clothing of men; and I can also speak with certainty, that blankets have conveyed it.

Have you had instances where contact with plague-patients has taken place, but no disorder has occurred?—Yes, I have known some such instances. On the first appearance of the disease, in the months of September, October, November, and December, when the disease was virulent, I knew of very few cases where the disease did not follow contact; but afterwards, when the most rigid precautions were taken, and towards what I have denominated the end of the season, and when the disease was in a mild form, people went sometimes together with impunity: a case of plague has been detected, and people in the same tent have not had it.

Were many taken with the plague together at the same time; simultaneously, as they call it?—I think not: the 160 fatal cases of plague in the Indian army were pretty equally divided among them the first four or five months after its appearance.

Of those afflicted with the plague, what proportion died?—I am sorry to say that, soon after the first part of the season, none recovered: indeed, the subjects of the first part of the season were mostly native troops, the Sepoys; and many unfavourable circumstances, along with the virulence of the disease, attended.

From what number of soldiers were those 165 taken?—The Indian army consisted of 7,886; of those 3,759 were Europeans, and 4,127 natives of India.

How many of the 165 were Europeans, and how many natives?—There died of the plague, of Europeans 38, and of natives of India 127.

When does the plague generally cease?—The inhabitants of Egypt have an idea that it ceases on St. John's day, the summer solstice.

To what do they attribute it?—The general belief is, that the extremes of heat and cold arrest the plague.

Do not the waters of the Nile overflow the Delta about June almost invariably?—The increase continues to that time, and there is a great deal of slime and mud thrown up on the banks of the river, from the low situation of the country. The Delta is a marshy low country; but the plague always commits as much devastation in Upper Egypt, where there is no marsh.

Does the plague subside suddenly?—No, it subsides gradually; from perhaps February or March till June.

Does the plague occur in Upper Egypt prior to Lower Egypt?—In descending from Upper Egypt, we understood, in its capital, that they had had what they termed *accidents*, before we came down; and that, in fact, the disease was never out of the country.

What time did you come down?—In June.

Where did you land?—At Kossier in the Arabian Gulf.

Do you recollect the month you landed there?—On the 16th May.

And from June to September was there no plague?—No plague in the British or Indian army; and it was confined there that year mostly to Rosetta.

To what cause do you attribute the suspension of the plague?—The natives of the country have an idea that extreme heat extinguishes the disease. It is a pretty general opinion, too, that the disease is never entirely extinct in the country, but that it is called into action by causes with which we are perhaps not very well acquainted.

Do you not consider that the exhalations of the slimy mud of the Delta may be the chief cause of the plague?—We know a specific disease caused by these exhalations, but very different from the plague.

Have you ever heard of plague in England?—I have heard of it in the history of the country.

What year?—The last appearance of plague was in 1665.

There has been no appearance of plague since?—I never understood there was.

Do you consider the disorder then to be the real plague of Egypt?—I have not read a description of it lately. I had no doubt, at the time I read of it, that it was the same.

Whose account are you speaking of?—Dr. Mead's.

Do you recollect that Dr. Sydenham was of opinion it was the plague?—I have not read of, or attended to, the subject of late years.

Have you known of any of the expurgators of goods in this country afflicted with the plague?—I never heard.

Do you not suppose that, if the plague had been brought in bales of goods to our quarantine establishments, some of the expurgators of goods must have been infected?—They must have run a great risk; the risk would be much diminished from the distance of time the things were packed or opened.

From their not having been infected, would it not be more likely to infer that no infection was brought in the bales?—I think it probably might; but there are many circumstances to be weighed between the present and the time the plague prevailed in England: improvements in the structures of the houses and towns; cleanliness, ventilation, and police regulations, are enforced in a degree unknown in the seventeenth century. Medical science stands now on different grounds; and we have acquired a knowledge of the disease, its treatment, and prevention. In Egypt, as well as in Turkey, many existing circumstances tend to the propagation of contagion, and rendering it more virulent. Were typhus fever to appear in those countries, it is difficult to conceive how it ever could be eradicated.

Do you consider the quarantine establishments of any use in this country, from the circumstance of the plague not having been in the country for so many years?—From the nature of fomites, I should consider the risk would be increased from having no quarantine. The risk in this country would be less, compared with a country where the intercourse was short, and by land, from the country where the plague raged.

From what undoubted truth of the contagion of the plague, that has come under your knowledge, do you found your idea that it is dangerous?—I should refer to my answers to former questions; and I could adduce many other instances that came under my knowledge in Egypt. A man was taken-up in Alexandria, and put in the main guard-house; the man, the day after, showed symptoms of plague, and was sent to the pest-house, and died of it: several of the guard, immediately after this, and prisoners, had the disease. Another instance I should like to mention: a Sepoy had the plague, and was sent to the pest-house at Aboukir; his wife insisted on accompanying him. The man died in a few days after he went there. The woman, who entreated not to be kept in any part of the pest-establishment, was sent to a hut at a distance, and a sentry placed over it; and, on the tenth day after her removal, she showed symptoms of the plague, and likewise died of it. I could adduce many similar instances.

